# The metabolic footprint of compromised insulin sensitivity under fasting and hyperinsulinemic-euglycemic clamp conditions in an Arab population

**DOI:** 10.1038/s41598-020-73723-8

**Published:** 2020-10-13

**Authors:** Anna Halama, Noor N. Suleiman, Michal Kulinski, Ilham Bettahi, Shaimaa Hassoun, Meis Alkasem, Ibrahem Abdalhakam, Ahmad Iskandarani, Tareq A. Samra, Stephen L. Atkin, Karsten Suhre, Abdul Badi Abou-Samra

**Affiliations:** 1 Department of Physiology and Biophysics, Weill Cornell Medicine-Qatar, Doha, Qatar; 2 Department of Internal Medicine, Qatar Metabolic Institute, Hamad Medical Corporation, Doha, Qatar; 3 Translational Research Institute, Academic Health System, Hamad Medical Corporation, Doha, Qatar; 4 Weill Cornell Medicine-Qatar, Doha, Qatar; 5 Royal College of Surgeons in Ireland, Busaiteen, Bahrain

**Keywords:** Lipidomics, Metabolomics, Pre-diabetes, Type 2 diabetes

## Abstract

Metabolic pathways that are corrupted at early stages of insulin resistance (IR) remain elusive. This study investigates changes in body metabolism in clinically healthy and otherwise asymptomatic subjects that may become apparent already under compromised insulin sensitivity (IS) and prior to IR. 47 clinically healthy Arab male subjects with a broad range of IS, determined by hyperinsulinemic-euglycemic clamp (HIEC), were investigated. Untargeted metabolomics and complex lipidomics were conducted on serum samples collected under fasting and HIEC conditions. Linear models were used to identify associations between metabolites concentrations and IS levels. Among 1896 identified metabolites, 551 showed significant differences between fasting and HIEC, reflecting the metabolic switch in energy utilization. At fasting, 336 metabolites, predominantly di- and tri-acylglycerols, showed significant differences between subjects with low and high levels of IS. Changes in amino acid, carbohydrate and fatty acid metabolism in response to insulin were impaired in subjects with low IS. Association of altered mannose and amino acids with IS was also replicated in an independent cohort of T2D patients. We identified metabolic phenotypes that characterize clinically healthy Arab subjects with low levels of IS at their fasting state. Our study is providing further insights into the metabolic pathways that precede IR.

## Introduction

Insulin resistance (IR) results from compromised cellular responsiveness to insulin, leading to impaired glucose homeostasis as well as altered glycogen and lipid storage^1^. The metabolic dysregulation driven by IR can progress to type 2 diabetes (T2D), which has become a major public health concern worldwide^1^. The increase in the prevalence of T2D was also recognized as a significant issue in the Arab region, where three Gulf Countries including Qatar, Kuwait and Saudi Arabia were found in the top ten for prevalence of T2D^2^. The onset of IR, quantified by impaired insulin sensitivity (IS), provides an early identification of subjects that are at increased risk of developing T2D. At this point, reversal of IR may still be possible through life style intervention^3,4^. However, impaired IS is asymptomatic and cannot be easily detected. Also, little is known about pathophysiological processes accompanying impaired IS at an early stage. It is therefore essential to obtain a deeper understanding of the pathophysiological processes preceding the development of full-blown IR.

The gold standard for measuring IS is the hyperinsulinemic-euglycemic clamp (HIEC) approach^5^. The rate of infused glucose under hyperinsulinemic conditions reflects the capacity of the body to uptake and metabolize glucose. The amount of infused glucose divided by body weight and time is expressed as the M-value [mg/kg/min], which is then used to define IR^6^. An M-value below 4.7 was suggested as a cut-off defining IR by Bergman et al.^6^, which was further revised to an M-value below 5.6 as a new cut-off by Tam et al.^4^ Conducting a HIEC is time consuming, invasive and thus not suitable for population screening^7^. Less invasive alternatives, such as the homeostasis model assessment of insulin resistance (HOMA-IR), which is based on single blood measurements of fasting glucose and insulin concentrations^8^, has been shown to be inaccurate on an individual-patient level, independently of its significant correlation with M-values^8^. Therefore, other indicators of IS could strongly benefit patients and healthcare systems.

Metabolomics is the measurement of ideally all relevant small molecules (metabolites) in a biological sample and can provide a comprehensive picture of the physiological status of the body. The metabolic composition of the body is defined as metabolic phenotype or metabotype. We and others have previously reported metabolic signatures of diabetes^9–16^. For example, alterations in the levels of certain circulating metabolites, including branched chain amino acids (BCAA), aromatic amino acids (AAA), as well as a number of lipids, were recognized as predictive markers of T2D in longitudinal studies^12,13^. Metabolic signatures of insulin resistance in plasma and muscle samples were also previously reported^11,14,16^. Elevated levels of BCAA, AAA, and alanine as well as phospholipids were identified as associated with insulin resistance determined by HOMA-IR^14^ Fasting plasma mannose concentrations also correlate with compromised IS in individuals with T2D^10^ and associate with insulin resistance^17^. In subjects divided into resistant and sensitive to insulin based on HIEC, alpha-hydroxybutyrate, 1-linoleoylglycrophosphatidylcholine and glycine were found to serve as metabolic markers of IR, based on their associations with M-values^18^. Although many metabolites have been associated with diabetes and its comorbidities, it remains to be determined whether early stages of compromised IS are also already reflected by the body’s metabolism. Additionally, the nature of the metabolic response to insulin infusion under glucose availability in subjects with compromised IS remains elusive, as the majority of studies so far have been conducted under fasting conditions. Furthermore, our current understanding of the metabolic dysregulations related to IR relies predominantly on studies conducted in a Caucasian population, which due to the genetic background and the life habits differ from an Arab population. Therefore, further understanding of metabolic dysregulations in an Arab population could lead to identification of novel signatures, which might serve as predictive markers enabling identification of subjects at risk of T2D.

Our objective here is to obtain a deeper insight into the metabolic features of the pathophysiological processes preceding IR and T2D in an Arab population. We used broad metabolic profiling using a commercial metabolomics and lipidomics provider (Metabolon Inc., Durham, NC) to identify alterations in body metabolism associated with compromised IS, both in a fasting state as well as under HIEC conditions to monitor the body’s response to insulin signaling under glucose availability. Furthermore, we attempted the replication of our findings in a cohort of T2D patients to assess which of the metabolic pathways affected by early stages of IS are also observed to be perturbed under conditions of advanced IR and T2D.

## Results

### Characteristics of study subjects

**Figure 1:**
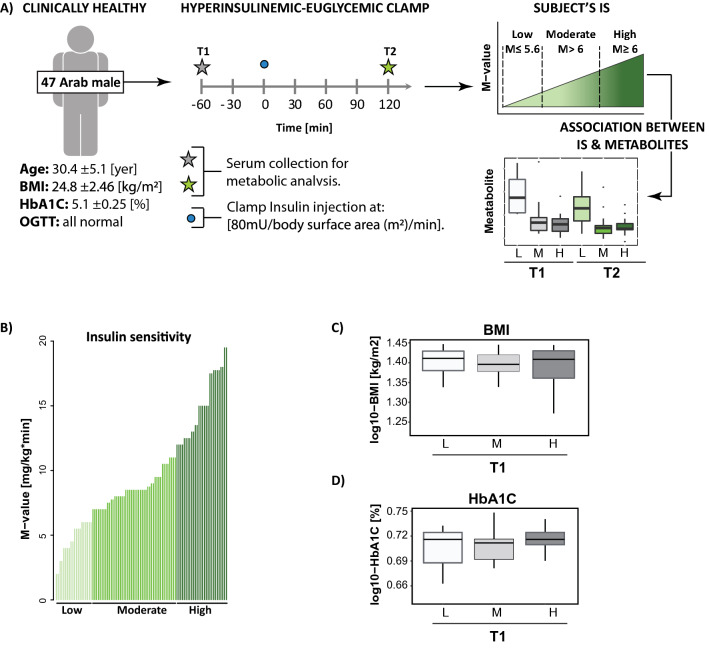
Subject characteristic. (**A**) Experimental design. 47 clinically healthy lean (body mass index (BMI) < 28) male subjects of Arab descent were enrolled for the study. The subjects underwent hyperinsulinemic-euglycemic clamp to determine their levels of insulin sensitivity (IS). The samples were collected before the clamp (T1) and at 120 min of the clamp (T2). Subjects were divided into the low (L), moderate (M) and high (H) responders to insulin based on their levels of IS depicted by the M-value. The samples collected at T1 and T2 were probed for the metabolic composition. Association analysis between level of metabolites and three levels of IS (L, M, H) was conducted at T1 and T2. (**B**) Levels of IS across the subject were determined at hyperinsulinemic-euglycemic clam and were depicted by M-values; the subjects were grouped into the low (M-value ≤ 5.6), moderate (M-value > 6) and high (M-value ≥ 12) responders to inulin, (**C**) association between BMI and IS. (**D**) Association between glycated hemoglobin (HbA1C) and IS levels. At T1 subjects levels of IS are depicted in gray scale from light grey indicating low IS into dark grey indicating high IS. At T2 subjects levels of IS are depicted in green scale from light green indicating low IS into dark grey indicating high IS.

This study included 47 [age 30.4 ± 5.1 (21–43)] healthy non-obese males of Arab descent (BMI 24.8 ± 2.46, range 16.9–28), normal HbA1C levels (HbA1C [%]: 5.1 ± 0.25, range 4.4–5.6), fasting glucose < 5.6 mmol/l, and blood glucose response to an oral glucose tolerance test (OGTT, 75 g) at 2 h below 7.8 mmol/l. We assessed the subjects’ IS using HIEC^5^. The experimental design is presented in Fig. 1A.

The HIEC revealed a wide range of the levels of IS as defined by the M-value [mg/kg/min] (M-value 9.69 ± 4.18, range 2–19.5) across the tested individuals (Fig. 1B). The distribution of the subjects’ M-values is presented in Supplementary Fig. 1. Following previous studies by Tam et al.^4^ individuals were grouped based on their level of IS into low (M-value ≤ 5.6), moderate (5.6 < M-value ≤ 12) and high (M-value > 12) responders to insulin. The study subjects were selected to have a BMI (below 28) and normal HbA1C (HbA1C % < 6). No significant association was detected between IS and these variables (Fig. 1C,D).

### Metabolic differences between the fasting state and hyperinsulinemic-euglycemic clamp reflect a metabolic switch in energy utilization

We conducted metabolome-wide profiling of serum samples collected at the fasting state (T1) and under euglycemic clamp conditions (T2) using two mass-spectrometry-based platforms, including the broad non-targeted metabolic profiling HD4 platform^19^ and the Lipdyzer complex lipid platform (CLP)^20^. Together, we identified 1896 molecules, 905 on the HD4 platform and 990 on the CLP platform, including 1697 named metabolites and 199 unnamed molecules (Supplementary Table 1).

**Figure 2:**
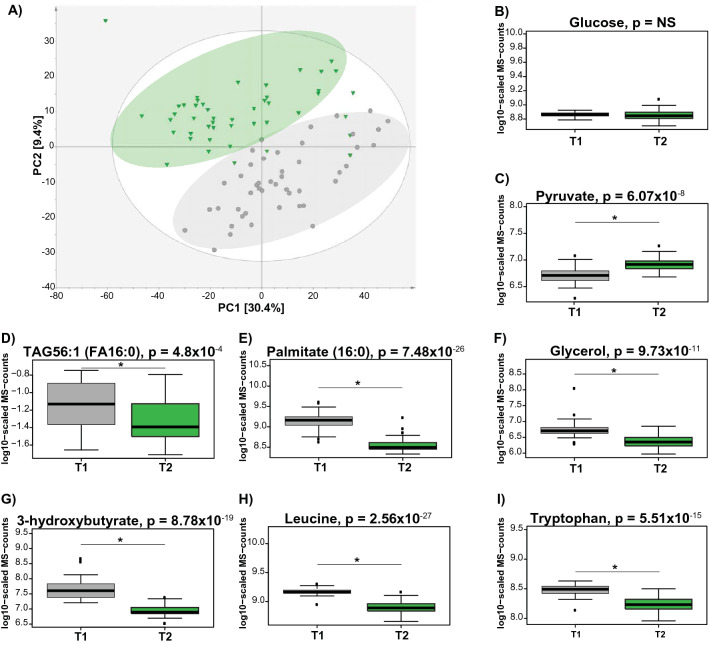
Impact of HIEC on body metabolism. (**A**) Principal component analysis (PCA) analysis reveals metabolic differences at the fasting state (T1) and under HIEC (T2). (**B**–**I**) Box plots present alternation patterns between fasting and HIEC conditions, and reflect body metabolism at fasting and fed state, respectively. Grey indicate the fasting state and green indicate HIEC. The p-value was defined by the statistical contrast between T1 and T2 and is depicted by “*”. TAG56:1(FA16:0): TAG—Triacylglycerol; 56—total number of carbons on all three fatty acid (FA) chains; (:1) one of the FA chains is unsaturated; and (FA16:0)—one FA chain consist of 16 carbons (16) and (:0)—is fully saturated.

We conducted a principle component analysis (PCA) including all detected metabolites to assess the impact of HIEC on overall body metabolism. We observed a clear separation between metabolic profiles between the fasting state (T1) and HIEC conditions (T2) (Fig. 2A).

**Figure 3:**
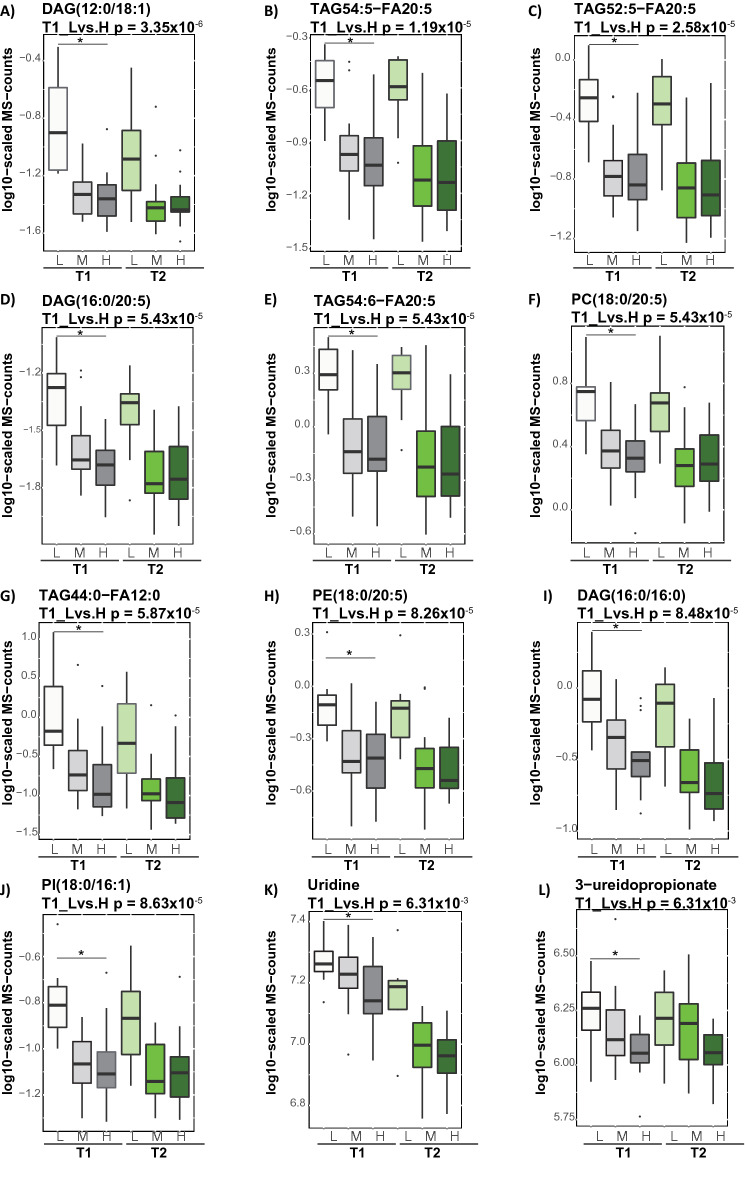
Triacylglycerols and diacylglycerols differentiate subjects with low IS level in the fasting state. Box blots presents top ten metabolites showing significant association with the levels of IS. The p-value was defined by the statistical contrast between estimated metabolite levels and IS [low (L) and high (H)] at subjects’ baseline (T1), depicted by “*”. Gray and green color gradient indicate metabolite levels after overnight fasting and hyperinsulinemic-euglycemic clamp, respectively. Light color tone indicate subjects with low IS and dark color tone indicate subjects with high IS.

The biochemical differences between postprandial and fasting state are reflecting switch in the energy utilization from glycolytic pathway to fatty acid oxidation^21^. Under fasting, the glucose level is maintained by hepatic gluconeogenesis, and the organism energetic needs are sustain by fatty acids (FA), released from triacylglycerols, catabolized in the process of beta-oxidation, ketone bodies and amino acids released from protein degradation^21^. We asked whether metabolic differences between fasting and HIEC would reassemble the metabolic switch, observed between fasting and postprandial state. Using a mixed linear model (see “Material and methods”), we identified 551 metabolites that showed significant alterations between fasting and HIEC at a false discovery rate (FDR)^22^ of 5% (Supplementary Table 2). The vast majority of the associated metabolites (514 out of 551) were elevated under fasting conditions in comparison to the HIEC state, including predominantly fatty acids, acylcarnitines, bile acids as well as molecules involved in branched chain amino acids (BCAA), aromatic amino acids (AAA), methionine and urea cycle metabolism. In contrast, molecules involved in carbohydrate metabolism and acyl cholines were elevated under HIEC conditions. Glucose was maintained at the same level at the fasting state and HIEC (Fig. 2B). Increase in the pyruvate level was observed under HIEC (Fig. 2C). Levels of TAG’s (TAG56:1 Fig. 2D, full list in Supplementary Table 2), fatty acids [Palmitate (16:0) Fig. 2E, full list in Supplementary Table 2], glycerol (Fig. 2F) and 3-hydroxybutyrate (Fig. 2G) decreased under HIEC conditions. The HIEC triggered a decrease in the level of amino acids including BCAA (Leucine Fig. 2H) and AAA (tryptophan Fig. 2I) (full list with altered amino acids in Supplementary Table 2). Interestingly, we even identified molecules from the clinical intervention: Subjects were given lidocaine as a local anesthetic prior to the clamp, and we found lidocaine and N-ethylglycinexylidide, which is a breakdown product of lidocaine, in most blood samples at T2 and very low levels at T1 (Supplementary Fig. 2).

These results indicate that metabolic differences between the fasting state and HIEC reflect the metabolic switch in the pathways of energy utilization from beta-oxidation of fatty acids under fasting to glucose utilization under glucose and insulin presence. This experimental setting provides unique and well defined conditions for further investigation of the impact of IS on body metabolism under conditions where fatty acids or glucose are utilized as primary energy source, as we show next.

### Elevated levels of triacylglycerols and diacylglycerols at baseline are the hallmark of compromised insulin sensitivity

We asked whether different levels of IS are impacting body metabolism in a fasting state, under conditions where fatty acids are utilized to fulfill the body’s energetic demand. The differences between the estimated metabolite levels in the three IS groups (low, moderate, and high) at the fasting-state (T1) were assessed (see “Material and methods”). We identified 336 metabolites showing FDR significant associations with the levels of IS (low, moderate, and high) at the fasting-state (T1) (Supplementary Table 3). The identified metabolites were predominantly lipids, such as TAGs (268 molecules with different lipid side chain compositions), diacylglycerols (29 molecules), and phosphatidylcholines (eight molecules), which were all elevated in subjects with low IS. The top ten molecules associated with IS are presented in Fig. 3; six of those lipid molecules contain an eicosapentaenoate (20:5n3) fatty acid chain. Only six out of 336 identified metabolites were not directly involved in lipid metabolism. Those metabolites were two nucleotides (uridine and 3-ureidopropionate), two amino acids involved in glutamate metabolism (alpha-ketoglutaramate and pyroglutamine) as well as two xenobiotics (betonicine and 2-hydroxyacetaminophen sulfate). The levels of urine and 3-ureidopropionate were elevated in subjects with low IS (Fig. 3).

Given that majority of metabolites associated with the levels of IS were triacylglycerols and diacylglycerols, we further tested, whether lipids monitored using clinical chemistry assays, such as total cholesterol (TC), total triacylglycerols (TG), high-density lipoproteins (HDL), and (LDL) low-density lipoproteins, differ across the subjects with low, moderate and high levels of IS. We observed significant differences in TC (p-value = 2.67 × 10^–3^), TG (p-value = 1. 78 × 10^–2^), and LDL (p-value = 2.06 × 10^–2^) but not HDL across the subjects with various IS levels (Supplementary Table 4). Next, we investigated the correlations between all metabolites and clinical chemistry readouts (TC, TG, HDL and LDL). The correlation analysis between metabolites and the levels of TC, TG, HDL, and LDL is presented in Supplementary Table 5. We found strong correlations (r > 0.7) between 24 metabolites and TC, between 66 metabolites and TG, as well as between 13 metabolites and LDL. The levels of TC correlated predominantly with cholesterol esters (CE), phosphatidylcholines (PC) and sphingomyelins (SM) consisting of long and very long chain fatty acids. The levels of LDL correlated with CE and SM. The levels of TG correlated with various TAGs and DAGs. We found that 71% of metabolites that correlated (r > 0.7) with the levels of TG overlapped with the metabolites significantly associated with the levels of IS. In contrast, none of the metabolites correlating with either TC or LDL levels overlapped with metabolites that associated with the levels of IS (Supplementary Fig. 3). Only a weak correlations (r < 0.5) were observed with HDL. These results indicate metabolic differences between subjects with low and high IS at the fasting state, which manifested in accumulation of triacylglycerols and diacylglycerols, and suggest a metabotype, which is specific to compromised IS, and is predominantly driven by dysregulated TG homeostasis.

### Metabolic dysregulation associated with early onset of compromised insulin sensitivity in response to insulin

We further tested whether a low IS would affect the metabolic responses to HIEC, under which glucose is utilized as a primary source of energy. First, we investigated the impact of HIEC on the metabolites already dysregulated at the fasting state. We found that under HIEC conditions, di- and tri-acylglycerols remain elevated in subjects with low IS, which suggest that those alterations are independent of the primary energy source.

Next, we identified 39 metabolites showing FDR significant association with the levels of IS (low, moderate, and high), defined by the statistical contrast between estimated metabolite levels in the three groups under HIEC (Table 1). The associated metabolites are from various metabolic pathways, including branched chain and aromatic amino acid metabolism, carbohydrate, and lipid catabolism. All identified amino acids, including the BCAAs isoleucine and leucine, the aromatic amino acids (tryptophan, phenylalanine, and tyrosine), as well as methionine and histidine were elevated in subjects with low IS. The elevated level of fatty acids [e.g. docosapentaenoate (22:5n3), eicosapentaenoate (20:5n3), dihomo-linolenate (20:3n3), myristoleate (14:1n5), and myristate (14:0)] in individuals with a low IS were also observed. Levels of almost all carbohydrates were lower in subjects with a low IS, with the exception of mannose, which is interesting as mannose was also inversely associated with a genetic higher diabetes risk at the GCKR locus^23^.

**Table 1:** List of metabolites showing FDR significant association with IS level under hyperinsulinemic-euglycemic clamp.

Metabolite	Sub-pathway metabolism	Beta	p-value	Beta (Rep)	p-value (Rep)
Histidine	Histidine	− 0.07	6.89 × 10^–3^	− 0.01	8.55 × 10^–1^
Isoleucine	Leucine, isoleucine and valine	− 0.13	1.53 × 10^–4^	− 0.29	2.72 × 10^–7^
Leucine		− 0.15	1.39 × 10^–5^	− 0.19	1.78 × 10^–4^
Methionine	Methionine and taurine	− 0.11	1.11 × 10^–3^	− 0.13	2.17 × 10^–3^
Phenylalanine	Phenylalanine	− 0.11	8.73 × 10^–5^	− 0.12	6.00 × 10^–3^
Tryptophan	Tryptophan	− 0.14	2.19 × 10^–4^	− 0.12	3.33 × 10^–3^
Tyrosine	Tyrosine	− 0.17	4.98 × 10^–5^	− 0.25	9.93 × 10^–7^
Erythronate	Aminosugar	0.12	7.77 × 10^–5^	− 0.05	2.26 × 10^–1^
Mannose	Fructose, mannose and galactose	− 0.26	1.99 × 10^–9^	− 0.42	1.28 × 10^–6^
Galactonate		0.71	4.45 × 10^–5^	0.28	4.17 × 10^–1^
Maltose	Glycogen	0.48	6.00 × 10^–8^	ND	ND
Pyruvate	Glycolysis	0.15	9.26 × 10^–3^	− 0.01	9.01 × 10^–1^
Arabinose	Pentose	0.25	1.51 × 10^–3^	− 0.02	7.31 × 10^–1^
Arabonate/xylonate		0.28	2.48 × 10^–5^	ND	ND
2-Hydroxystearate	Fatty acid, monohydroxy	− 0.12	9.66 × 10^–3^	0.02	7.48 × 10^–1^
Myristoleate (14:1n5)	Long chain fatty acid	− 0.41	2.06 × 10^–3^	− 0.55	3.20 × 10^–3^
10-Heptadecenoate (17:1n7)		− 0.31	4.83 × 10^–3^	− 0.36	2.73 × 10^–3^
Myristate (14:0)		− 0.21	1.30 × 10^–2^	− 0.14	1.53 × 10^–1^
FFA(18:1)		− 0.19	2.31 × 10^–3^	ND	ND
FFA(18:2)		− 0.18	8.41 × 10^–3^	ND	ND
Oleate/vaccenate (18:1)		− 0.33	6.89 × 10^–3^	ND	ND
Caprate (10:0)	Medium chain fatty acid	− 0.25	2.58 × 10^–3^	− 0.07	2.63 × 10^–1^
Laurate (12:0)		− 0.23	4.13 × 10^–3^	− 0.06	5.06 × 10^–1^
Linolenate	Polyunsaturated fatty acid	− 0.31	5.11 × 10^–3^	− 0.34	9.31 × 10^–3^
Adrenate (22:4n6)		− 0.34	3.80 × 10^–3^	− 0.20	1.60 × 10^–1^
Docosapentaenoate (DPA; 22:5n3)		− 0.24	1.01 × 10^–3^	− 0.09	3.53 × 10^–1^
Eicosapentaenoate (EPA; 20:5n3)		− 0.36	1.49 × 10^–4^	0.05	5.81 × 10^–1^
Dihomo-linolenate (20:3n3)		− 0.23	5.44 × 10^–3^	− 0.06	3.91 × 10^–1^
PC(16:0/12:0)	Phosphatidylcholine	− 0.16	9.62 × 10^–4^	ND	ND
TAG46:1-FA14:1	Triacylglycerol	− 0.37	8.14 × 10^–6^	ND	ND
Uridine	Pyrimidine	− 0.21	3.31 × 10^–6^	− 0.12	1.78 × 10^–3^
γ-Glutamylmethionine	Gamma-glutamyl amino acid	− 0.13	3.30 × 10^–3^	− 0.15	6.29 × 10^–2^
γ-Glutamylphenylalanine		− 0.14	6.18 × 10^–3^	− 0.14	1.37 × 10^–2^
X-24813	Unknown	− 0.09	5.21 × 10^–4^	ND	ND
X-24455		− 0.14	1.14 × 10^–2^	ND	ND
X-23369		− 0.24	1.64 × 10^–3^	ND	ND
Lactobacillic acid	Bacterial/Fungal	− 0.30	1.24 × 10^–2^	ND	ND
Gluconate	Food component/plant	0.52	7.25 × 10^–6^	0.18	5.49 × 10^–3^
Tartarate		0.36	9.46 × 10^–5^	ND	ND

ND indicate not detected metabolites in replication cohort. The p-value was defined by the statistical contrast between estimated metabolite levels and IS (high and low) under hyperinsulinemic-euglycemic clamp (T2). Beta, reflects the directionality of the changes relative to the subjects with high IS. Beta (rep) and p-value (rep) indicate coefficient and p-value values calculated in replication cohort, respectively.

These results reveal metabolic dysregulations associated with low IS under HIEC, and indicate metabolic responses, which are impaired under the insulin action and glucose availability, further suggesting its potential involvement in progression toward IR and T2D.

### Impaired metabolic response to insulin in clinically healthy subjects replicates in T2D patients

**Figure 4:**
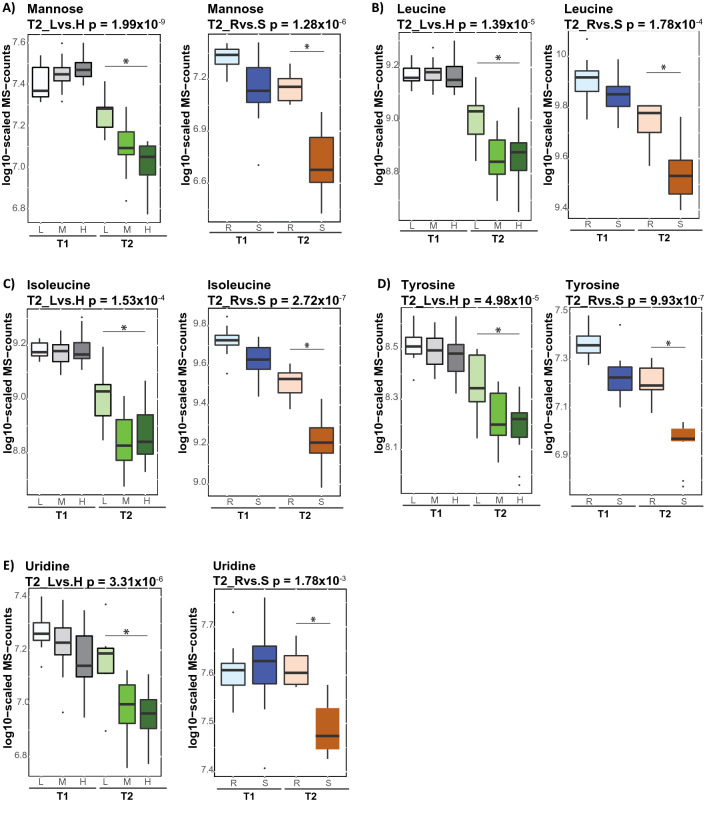
Different metabolic responses to insulin under hyperinsulinemic-euglycemic clamp in clinically healthy subjects replicate in independent cohort consist of T2D (**A**–**E**). Box blots presents metabolites showing FDR significantly different responses to HIEC, associated with the levels of IS. The p-value was defined by the statistical contrast under hyperinsulinemic-euglycemic clamp (T2), between estimated metabolite levels and IS [low (L) and high (H)] in clinically healthy or between estimated metabolite levels and insulin-resistance/insulin-sensitive group in replication cohort of T2D. Gray and green color gradient indicate metabolite levels after overnight fasting and hyperinsulinemic-euglycemic clamp, respectively. Light color tone indicate subjects with low IS and dark color tone indicate subjects with high IS. The replication cohort is depicted by blue (fasting-state) and orange (at HIEC) color. Light blue—insulin resistant (R), dark blue—insulin-sensitive (S), light orange—insulin resistant (R), dark orange—insulin sensitive (S).

Lastly we tested whether the metabolic dysregulations observed under HIEC in clinically healthy subjects with low IS might be implicated in the future progression toward IR and T2D. We conducted replication in an independent cohort of 17 subjects, which included healthy controls (seven subjects) and T2D (ten subjects). The HIEC was performed as we previously reported^24^ and the M-values [mg/kg/min] were (M-value: 4.78 ± 2.81, range 0.75–10.8). The subjects were grouped into insulin sensitive (M-value > 4.7) and insulin resistance (M-value ≤ 4.7) based on the conservative definition of Bergman et al.^6^ The metabolic profiling was conducted using a non-targeted metabolomics platform after overnight fasting (T1) and 120 min. at HIEC (T2). We identified 22 metabolites showing FDR significant association with IS in the insulin-sensitive and insulin-resistant groups under HIEC. Among the metabolites showing FDR significant association with IS in clinically healthy subjects of Arab descent, we replicated the same trend at the nominally significant p-value for almost all amino acids apart of histidine (Table 1). The association of mannose, isoleucine, leucine, tyrosine, and uridine with IS in clinically healthy subjects was replicated at the FDR significance level in the T2D patients of this independent cohort (Fig. 4). In the replication cohort, we observed an FDR significant association of mannose (p-value = 2.45 × 10^–3^) and tyrosine (p-value = 3.01 × 10^–3^) with IS at the baseline (T1). Among the lipids, polyunsaturated fatty acids (PUFA) were not replicated.

These results further suggest that impaired responses of mannose, branch chain amino acid, tyrosine and uridine to insulin, observed in clinically healthy subjects with low IS, could be considered as key metabolic dysregulations, which together with accumulated lipid triggers progression toward IR and T2D phenotype.

## Discussion

In the present work, we identified specific metabotypes in Arab men that describe apparently healthy subjects with low IS under fasting conditions. Our study illustrates how the body’s metabolism in individuals with low IS responds to insulin under glucose availability, and provides further insight into the metabolic pathways preceding IR and T2D.

There is a growing body of evidence that suggests a role for metabolic dysregulations in the development of IR and T2D^12,15,25–27^. However, the current knowledge on the metabolic dysregulations driven by T2D or IR emerged predominantly from studies on a Caucasian cohorts investigating subjects in the fasting state. In contrast, we monitored apparently healthy subjects of Arab descent at the fasting state and under HIEC conditions. We found that metabolic signatures, including BCAA, AAA, as well as mannose, reported previously as predictors of T2D or IR in longitudinal studies^10,12,13,17^, appeared as significantly associated with the levels of IS under HIEC, but not at fasting state. At fasting state metabolism related to insulin signaling is suppressed and catabolic processes are activated to fulfill the energy needs of the body^21^. Thus, our observation suggests that subjects investigated in our study were captured at early stages, preceding IR, as the metabolic signatures previously related to T2D or IR, were revealed only under acute responses to insulin. Therefore, it could be reasoned that metabolites showing association with the levels of IS at fasting state would be a sensitive indicators of subjects at risk of IR and T2D.

Our study demonstrates that elevated levels of di- and tri-acylglycerols as well as uridine are the hallmark of subjects with low IS at the fasting state, suggesting elevated metabolic fluxes through the TAG cycle. This observation is in good agreement with a previous longitudinal study showing an association between elevated levels of triglycerides and the risk of T2D in young men^28^. However, the previous study reported on total triglyceride, and were not resolving individual triglyceride structures. Another study conducted in a Chinese cohort identified 34 different tri-acylglycerols as predictive markers for the development of T2D^29^. We found that 18 out of these 34 diabetes-associated triglycerides also showed significant associations with the levels of IS in our study, further supporting a role of these lipids in the development of IR, and that this association is likely independent of the investigated population. Ectopic deposition of lipids in insulin-targeted tissues and elevated levels of diacylglycerols and triacylglycerols are thought to be important contributors to the pathogenesis of IR in obese and T2D patients^11,12,30^. Hence, high levels of circulating di- and tri-acylglycerols in clinically healthy subjects with a low IS, observed in our study, might be an indicator for individuals that progress towards IR and T2D. The elevated level of TAG in the blood indicate suppressed beta oxidation of fatty acids, which would trigger a TAG flux to balance the FA blood levels. Indeed, an increased rate of beta oxidation of fatty acids was shown to protect against FA induced IR^31,32^, which supports this reasoning.

The results of our study, however, did not replicated metabolites previously reported as markers of IR, including alpha-hydroxybutyrate, 1-linoleoylglycrophosphatidylcholine and glycine, based on their associations with M-values, although all three molecules were detected^18^. However, the previous studies were conducted in a Caucasian population and were limited to metabolic profiling, in contrast to our study in which global metabolic and complex lipidomics profiling were measured in an Arab cohort. Furthermore, subjects investigated in the previous study could have more advanced IR, especially since alpha-hydroxybutyrate, was previously reported as a selective metabolite biomarker of impaired glucose tolerance^33^. Noteworthy, the glucose tolerance of subjects monitored in our study was normal (at 2 h less than 7.8 mmol/l), further supporting healthier phenotype of the subjects, and thus lack of alteration in alpha-hydroxybutyrate, 1-linoleoylglycrophosphatidylcholine and glycine.

The replication approach of the metabolic responses under HIEC, which we undertook in an independent cohort of individuals with T2D, further revealed that mannose, isoleucine, leucine, tyrosine and uridine metabolism are affected in a similar manner in T2D. The interplay between lipids and BCAA in the progression of insulin resistance has been suggested before^27^. These observations suggest a limited capacity for the utilization of amino acids and are potentially due to a lipid overload that we showed is prevalent in the fasting state. Importantly, the BMI range of all of our study subjects was below^28^. Therefore, obesity is not the driving factor of the observed level of IS in our cohort, in agreement with previous report suggesting the personal fat threshold as a contributor to the progression towards T2D in non-obese subjects^34^.

Elevated levels of mannose have been identified as a marker of IR and T2D in multiple studies^9,10,17,35^. In the previous study, it was suggested that plasma mannose is a product of glycogen breakdown catalyzed by liver hexose 6-phosphate, which enables conversion of mannose-6-phosphate into mannose, and its accumulation^36^. Additionally, significant associations between mannose (p-value = 1.29 × 10^–77^) and genetic variation in GCKR (e.g. with SNP rs1260326) have previously been reported^23^. Interestingly, GCKR encodes the glucokinase regulatory protein (GKRP), which is a hepatocyte-specific inhibitor of the glucose-metabolizing enzyme glucokinase (GCK), previously associated with different metabolic traits including T2D, non-alcoholic fatty liver, fasting insulin and total cholesterol levels^37^. Association between the minor allele of the GCKR SNP rs1260326 (Pro446Leu) and higher fasting serum TG concentration as well as lower glucose concentration was previously reported in population studies^38–40^. Thus, elevated levels of mannose observed in the subject with low IS could suggest genetic variation in GCKR, which would require further investigation.

We previously reported that intensive lifestyle intervention leads to diabetes remission in over 60% of participants and normoglycaemia in over 30% of participants, as well as a decrease in total TG and an increase in HDL levels^3^. Thus, it could be reasoned that sensitivity to insulin might potentially be restored by healthy lifestyle and diet choices, which have a positive impact on body metabolism by normalizing lipid levels. Additionally, medications lowering lipid content were shown to improve sensitivity to insulin^41^. However, identification of subjects at risk of IR for whom early lifestyle intervention offers clear health benefits is challenging, given the asymptomatic nature of the disease. Moreover, standard methods such as HIEC, which enables the identification of subjects with low IS, are not practical enough to be applied in routine clinical practice^7^. Therefore, di- and tri-acylglycerols determined in our study could serve as potential metabolic markers for identification of individuals with subclinical IR. Importantly, the subjects participated in our study were defined as healthy based on the clinical parameters including OGTT and HbA1C. Additionally the average levels of total TG, measured with clinical chemistry assay, were normal (TG < 1.7 mmol/l), although differed significantly across the groups. Thus, standard clinical tests other than HIEC would be insufficient to identify subjects with compromised IS at risk of IR and T2D, which further support an emerging need for the implementation of a metabolomics approach into the clinic. Given that newborn screening for inborn errors of metabolism, deploying targeted metabolomics, is routinely conducted in the clinic^42^, translation of our findings for clinical purposes could be envisioned.

Our study, however, also has several limitations that would require additional investigation. First, our study was conducted in small cohort consisting only of Arab male subjects. Although, we limited this study to a homogenous group of male individuals to reduce variability and increase statistical power, future studies would require a bigger cohort to include female subjects, given gender-specific differences in metabolism^43^. Second, we showed metabolic associations with the levels of IS, but our study needs further replication in a longitudinal setting, which would be required for determination and validation of predictive markers of IR. Third, nutrition as well as lifestyle choices may impact the lipid metabolism^44^, but were not controlled for in our study and should be taken under consideration in future investigations. Fourth, our study suggests a role for diacylglycerols and triacylglycerols in processes preceding IR. Nevertheless, a study showing reversal of such metabotypes along with improvement of the M-values would be required as further proof for this reasoning.

## Conclusions

In summary, we described the metabolic footprint of compromised IS in clinically healthy subjects of Arab descent at two different conditions, namely at the fasting state, where fatty acids are utilized as primary source of energy, and under HIEC conditions, where glucose is utilized as an energy source under insulin action. We found that lipid metabolism was dysregulated in subjects with low IS, and that accumulation of di- and tri-acylglycerols could be considered as the hallmark of compromised IS as an early marker of on-setting IR. Additionally, we showed that some impaired metabolic responses to insulin are also seen further in the progression toward T2D. Our study provides further insight into the pathophysiological processes preceding IR and identifies metabolites that could in the future be monitored to screen asymptomatic subjects which are at risk of developing IR and T2D.

## Material and methods

### Subject characteristic

All experiments and procedures conformed to the 1975 Declaration of Helsinki. All experiments were conducted at the Qatar Metabolic Institute (Doha, Qatar) under approval by Institutional Review Board protocol (14224/14) IRB of Hamad Medical Corporation. All study participants gave their signed informed consent.

Male individuals of Arab descent with no known disease or pathology were recruited and screened by physical examination and standard blood biochemistry (n = 152). Inclusion criteria were: (1) Age > 18 and < 60, and (2) BMI ≤ 28 and BMI ≥ 16, and (3) normal complete blood count (CBC) [White blood cells (WBC): 4000–11,000/µl of blood, Red blood cells (RBC): 4.7 × 10^6^–6.1 × 10^6^/µl of blood, and Hemoglobin: 13.5–17.5 g/dl], and (4) normal blood chemistry [Alanine aminotransferase test (ALT): 7–56 IU/l, Aspartate aminotransferase test (AST): 10–40 IU/l, Alkaline Phosphatase (ALP): 20–140 IU/l, creatinine 0.7–1.2 mg/dl, Thyroid-Stimulating Hormone (TSH): 0.4–4.0 mIU/l, and normal Free Thyroxine (T4): 0.8–2.8 ng/dl], and (5) fasting glucose < 5.6 mmol/l, and (6) HbA1c: 4–5.6%, and (6) normal glucose response to OGTT (at 2 h less than 7.8 mmol/l), and (7) normal electrocardiogram (ECG), monitored by normal duration (interval) of the QRS complex 0.08–0.10 s, and (8) not on any medication. A total of 62 subjects were considered apparently healthy as they fulfilled all inclusion criteria and were submitted to an HIEC test. Blood samples were collected before (T1 = 0 min) and at the end of insulin stimulation (T2 = 120 min). Material for metabolomics and lipidomics analyses was obtained from 47 subjects, for which the blood was collected in the suitable for metabolomics tubes.

### Study design

The subjects enrolled for the study underwent (1) an initial screening that included clinical examination and fasting blood analysis for electrolytes, glucose, liver enzymes, lipid profile, complete blood count (CBC) and thyroid-stimulating hormone (TSH), urinalysis, and electrocardiogram (ECG); (2) a 75 g oral glucose tolerance test (OGTT); (3) a whole body DEXA scan to determine regional fat and fat-free mass and the lean body to assess insulin-stimulated glucose disposal rates during the insulin clamp; (4) a muscle biopsy; and (5) a hyperinsulinemic-euglycemic clamp (HIEC).

### Hyperinsulinemic-euglycemic clamp challenge

Subjects were admitted to the research study unit at 7 a.m., the site for muscle biopsy was identified and 10 ml of Xylocaine 2% (Braun, Melsungen, Germany) was applied subcutaneously and deeply into the muscle. An HIEC challenge was then conducted as previously described^24^. Briefly, the insulin infusion (100 IU/ml insulin solution, Actrapid) rate was constant throughout the hyperinsulinaemic-euglycemic clamp at [80 mU/body surface area (m^2^)/min]. The body surface area (m^2^) was calculated as previously described [0.007184 × (height(cm)^0.725^) × (weight(kg)^0.425^)]^45^. The blood glucose level was modulated by the infusion of 20% dextrose, which was adjusted every 5 min to achieve the targeted, stable blood glucose level of 90 mg/dl (5 mmol/l) for 120 min under the HIEC. The metabolizable glucose values (M-value) [mg/kg/min] was calculated from the infused exogenous glucose during the second hour of the insulin clamp divided by body mass and time (1 h).

### Sample collection

Venous blood samples were collected at two time points: (1) after overnight fasting, prior to the hyperinsulinemic-euglycemic clamp and (2) at 120 min of hyperinsulinemic-euglycemic clamp. The blood samples were collected in serum separator tubes, centrifuged at 1200 × *g* at 4 °C for 10 min, aliquoted to avoid any further freeze–thaw cycles and then stored at − 80 °C until the time of measurements.

### Metabolite measurements

The Metabolon HD4 metabolomics platform and the ABI Sciex Lipidyzer complex lipid platform (CLP), operated by Metabolon, were used for measurements. For each platform a separate aliquot was submitted for 47 individuals probed at two time points: (1) after an overnight fast (T1) and (2) at 120 min of the HIEC (T2). Both platforms have been extensively used in metabolomics experiments before, including our own lab^19,20,23,46,47^. We therefore have only briefly summarize the main features of these platforms below. Obtained metabolic data is provided in Supplementary Table 6.

#### Non-targeted metabolic profiling (metabolon HD4 platform)

The samples were processed as previously described^48^. The samples extracts were divided into equal fractions, dedicated for the measurements at different analytical platforms, and were evaporated under nitrogen stream [TurboVap (Zymark)], followed by reconstitution in four different solvents as previously described^48^. The measurements were all conducted using Waters ACQUITY ultra liquid chromatography (UPLC) and Thermo Scientific Q-Exactive high resolution/accurate mass spectrometer interfaced with a heated electrospray ionization (HESI-II) source and Orbitrap mass analyzer operated at 35,000 mass resolution as previously described^48^. The scan range varied between methods and covered 70–1000 m/z. The components were identified based on the extracted raw data using Metabolon’s proprietary software. Metabolon’s compound annotations and data have been independently validated by many previous studies, including several genetic association studies by our own group^23,47^.

#### Lipidomic profiling (ABI Sciex Lipidyzer CLP platform)

The samples were extracted using a butanol:methanol (BUME) mixture (3:1) followed by two-phase extraction into 300 µl heptane:ethyl acetate (3:1) using 300 µl 1% acetic acid as a buffer, in the presence of internal standards, as previously described^49^. The extracts were dried under nitrogen flow and reconstituted in ammonium acetate dichloromethane:methanol. The extracts infusion was performed on a Shimadzu LC with nano PEEK tubing. The samples were analyzed in both positive and negative mode electrospray using Sciex SelexIon-5500 QTRAP. The molecules were detected in MRM mode with a total of more than 1100 MRMs. Individual lipid species were quantified by the ratio of the signal intensity of each target compound to that of its assigned internal standard, followed by the multiplication of the concentration of internal standard added to the sample. Lipid class concentrations were calculated from the sum of all molecular species within a class, and fatty acid compositions were determined by calculating the proportion of each class comprised by individual fatty acids. The CLP platform quality and accuracy was evaluated in our previous study^46^.

### Statistical data analysis

Data analysis was performed using R studio (R version 3.5.3) using the R-package limma^50^ through our in‐house developed tool “autonomics” (freely available at https://github.com/bhagwataditya/autonomics). Metabolite levels were scaled by run-day medians and log-transformed. The levels of insulin sensitivity were defined as high, moderate, and low, based on the M-values calculated from HIEC (high ≥ 12, moderate > 6 and low ≤ 5.6). We then coded the interaction of three conditions reflecting insulin sensitivity (high, moderate, low) and two time points (baseline and hyperinsulinemic-euglycemic clamp) as six different subgroups. Then, we fitted the general linear model log10(exprs) ~ 0 + subgroup|subject_id, and investigated the following contrasts:

(A) (high_2h + medium_2h + low_2h)/3—(high_0h + medium_0h + low_0h)/3; (B) high_0h − low_0h; (C) high_2h − low_2h; and high_2h − high_0h − low_2h − low_0h, using the R package limma^50^. The p-values (0.05) were corrected for multiple testing using false discovery rate (FDR) correction^22^.

An online tool https://bioinformatics.psb.ugent.be/webtools/Venn/ was used to create Venn diagrams.

## Supplementary information

Supplementary Information.

Supplementary Figure 1.

Supplementary Figure 2.

Supplementary Figure 3.

Supplementary Table 1.

Supplementary Table 2.

Supplementary Table 3.

Supplementary Table 4.

Supplementary Table 5.

Supplementary Table 6.
