# Supplementary material for: The metabolic footprint of compromised insulin sensitivity under fasting and hyperinsulinemic-euglycemic clamp conditions in an Arab population
Source: Sci Rep. 2020 Oct 13;10:17164. doi: 10.1038/s41598-020-73723-8 (PMC7555540; doi:10.1038/s41598-020-73723-8)
Supplement: Supplementary file 1 — Supplementary Information. [file 41598_2020_73723_MOESM1_ESM.docx]

**Supplementary Materials.**

**Supplementary Table 1.** List of measured metabolites.

**Supplementary Table 2.** Metabolites showing significant alterations under HIEC.

**Supplementary Table 3.** Metabolites showing FDR significant association with the levels of IS (low, moderate and high) at the fasting-state (T1).

**Supplementary Table 4.** Average [+/- s.d.] values of different lipid molecules levels [mmol/L] measured using clinical chemistry assays by IS group. P-values correspond to ANOVA-test.**Supplementary Table 5.** Correlations between metabolites and levels of TC, TG, LDL, and HDL, determined with clinical chemistry assys.

**Supplementary Table 6.** Raw metabolomics data. The metabolites intensities (raw area counts) normalized for a single day run.

**Supplementary Figures.**

**Supplementary Figure 1.** Histogram of subjects M-values distribution.

**Supplementary Figure 2.** Box plot of molecules that reflected the clinical intervention. Grey and green indicate T1 and T2 respectively.

**Supplementary Figure 3.** Venn diagram showing the overlap between metabolites associated with IS levels at baseline (T1) and metabolites correlating (r > 0.7) with the levels of TC, TG, HDL, and LDL measured with clinical chemistry assays. The correlations between HDL and metabolites were weak r < 0.5 and not included in the analysis.
