# Supplementary material for: The metabolic footprint of compromised insulin sensitivity under fasting and hyperinsulinemic-euglycemic clamp conditions in an Arab population
Source: Sci Rep. 2020 Oct 13;10:17164. doi: 10.1038/s41598-020-73723-8 (PMC7555540; doi:10.1038/s41598-020-73723-8)
Supplement: Supplementary file 8 — Supplementary Table 4. [file 41598_2020_73723_MOESM8_ESM.docx]

**The metabolic footprint of compromised insulin sensitivity under fasting and hyperinsulinemic-euglycemic clamp conditions in an Arab population.**

**Short title: Metabotype of compromised insulin sensitivity in an Arab population.**

Anna Halama^1#^, Noor N. Suleiman^2#^, Michal Kulinski^3^, Ilham Bettahi^2,3^, Shaimaa Hassoun^2^, Meis Alkasem^2,3^, Ibrahem Abdalhakam^2^, Ahmad Iskandarani^2,3^, Tareq A. Samra^2,3^, Stephen Atkin^4,5^, Karsten Suhre^1^*, Abdul Badi Abou-Samra^2,4^*

^1^Department of Physiology and Biophysics, Weill Cornell Medicine – Qatar, Doha, Qatar.

^2^Qatar Metabolic Institute, Department of Internal Medicine, Hamad Medical Corporation, Doha, Qatar.

^3^Translational Research Institute, Academic Health System, Hamad Medical Corporation, Doha, Qatar.

^4^ Weill Cornell Medicine-Qatar, Doha, Qatar.

^5^Royal College of Surgeons in Ireland, Bahrain

**Supplementary Table 4.** Average [+/- s.d.] values of different lipid molecules levels [mmol/L] measured using clinical chemistry assays by IS group. P-values correspond to ANOVA-test.

|  | **Low** | **Moderate** | **High** | **p-value** |
| --- | --- | --- | --- | --- |
| **TC** | 5.53 [±1.38] | 4.34 [±0.45] | 4.40 [±0.86] | 2.67x10-3* |
| **TG** | 1.39 [±0.23] | 0.86[±0.22] | 1.06[±0.09] | 1.78x10-3* |
| **LDL** | 3.57[±1.23] | 2.71[±0.36] | 2.83[±0.61] | 2.06x10-3* |
| **HDL** | 1.33 [±0.10] | 1.23[±0.08] | 1.16[±0.05] | NS |

“*” indicate significant differences across the IS levels defined as low, moderate and high.
